# Rapid Carbon Formation from Spontaneous Reaction of Ferrocene and Liquid Bromine at Ambient Conditions

**DOI:** 10.3390/nano10081564

**Published:** 2020-08-09

**Authors:** Nikolaos Chalmpes, Iosif Tantis, Aristides Bakandritsos, Athanasios B. Bourlinos, Michael A. Karakassides, Dimitrios Gournis

**Affiliations:** 1 Department of Materials Science & Engineering, University of Ioannina, 45110 Ioannina, Greece; chalmpesnikos@gmail.com (N.C.); mkarakas@uoi.gr (M.A.K.); 2 Regional Centre of Advanced Technologies and Materials, Faculty of Science, Palacký University in Olomouc, Šlechtitelů 27, 779 00 Olomouc, Czech Republic; iosif.tantis@upol.cz (I.T.); a.bakandritsos@upol.cz (A.B.); 3 Physics Department, University of Ioannina, 45110 Ioannina, Greece

**Keywords:** ferrocene, bromine, carbon, rapid synthesis, ambient conditions

## Abstract

Herein, we present an interesting route to carbon derived from ferrocene without pyrolysis. Specifically, the direct contact of the metallocene with liquid bromine at ambient conditions released rapidly and spontaneously carbon soot, the latter containing dense spheres, nanosheets, and hollow spheres. The derived carbon carried surface C-Br bonds that permitted postfunctionalization of the solid through nucleophilic substitution. For instance, treatment with diglycolamine led to covalent attachment of the amine onto the carbon surface, thus conferring aqueous dispersability to t he solid. The dispersed solid exhibited visible photoluminescence under UV irradiation as a result of surface passivation by the amine. Hence, the present method not only allowed a rapid and spontaneous carbon formation at ambient conditions, but also surface engineering of the particles to impart new properties (e.g., photoluminescence).

## 1. Introduction

Carbon is an enchanting element that plays a central role in materials science and chemistry [1]. It is generally obtained from organic compounds by pyrolysis at elevated temperature in order to provide the necessary energy needed to extract the carbon atoms from its compounds (i.e., an energy consuming process). Alternatively, carbon can be obtained by non-pyrolytic methods, such as chemical vapor deposition or hydrothermal, which is also energy consuming (e.g., both require a high temperature to operate synthesis). However, is it possible to make carbon at room temperature without spending energy? The answer is yes. An old yet classic example refers to the exothermic carbonization of sugar by concentrated sulfuric acid. Most recently, hypergolic reactions were successfully utilized from our group for the spontaneous and fast preparation of a variety of functional carbon materials, such as carbon nanosheets, crystalline graphite, graphitic carbon nitride, photoluminescent carbon dots, and fullerols, at ambient conditions [2,3,4,5]. By definition, in hypergolic reactions two reagents react immediately and energetically upon contact. To that aim, an organic material serving as the carbon source and a powerful oxidizer were simply mixed at room temperature and atmospheric pressure to react exothermically towards the carbon phase formation. It was these results that further urged us to continue searching for new reaction schemes for the production of carbon at ambient conditions in an energy-liberating manner.

Ferrocene Fe(C_5_H_5_)_2_, an organometallic compound consisting of two cyclopentadienyl rings bonded by divalent iron (II), is a versatile precursor towards carbon materials by pyrolysis (i.e., an energy consuming process) [6,7,8]. Fewer works also refer to hydrothermal carbonization of ferrocene, which, however, is an energy consuming process as well [9]. Herein, we present an interesting case of carbon formation from ferrocene without pyrolysis or hydrothermal treatment, which is merely based on the spontaneous and rapid reaction of the metallocene with liquid bromine at ambient conditions. The reaction led to carbons of various morphologies (dense or hollow spheres and nanosheets) that contained reactive C–Br groups on the surface. Subsequent nucleophilic substitution of the bromine atoms by amines gave dispersible derivatives that fluoresced in the visible region upon UV irradiation. Such a property was absent from the parent solid, thus demonstrating the impact of functionalization on the physical properties of the derived carbon. The reaction of ferrocene with liquid bromine offers new pathways to carbon that is enriching to the synthesis tools described above. In addition, since the reaction is exothermic *(vide infra)* the present preparative method should be considered as energy liberating rather than energy consuming.

## 2. Materials and Methods

**Figure 1:**
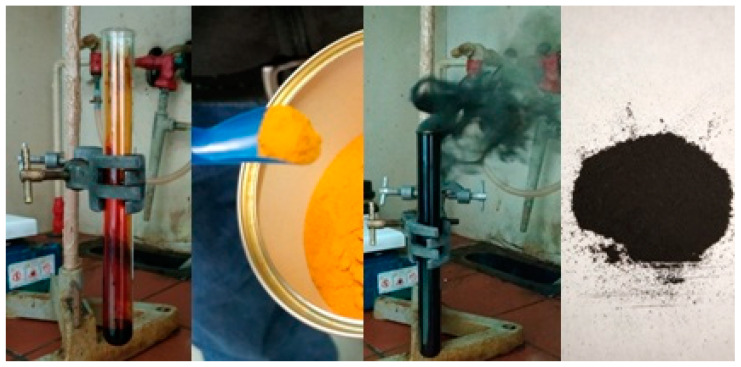
Addition of ferrocene to liquid bromine rapidly led to an exothermic reaction that released carbon soot partly deposited on the walls of the test tube. After collecting and washing the carbon residue, a fine black powder was obtained.

Synthesis was conducted in a fume hood. A glass test tube (diameter: 1.5 cm; length: 15 cm) was charged with 1 mL liquid bromine (99% Merck KGaA, Darmstadt, Germany) followed by the gradual addition of 1 g ferrocene (fine powder, 98% Sigma–Aldrich, St. Louis, MO, USA) in portions of 250 mg. Ferrocene and bromine reacted rapidly and exothermically upon contact to release carbon soot partly deposited on the walls of the test tube. The product was scratched off the walls of the test tube and then thoroughly washed with acetone (≥99% Merck KGaA, Darmstadt, Germany), water, tetrahydrofuran (THF, ≥99%, Merck KGaA, Darmstadt, Germany), and chloroform (≥99% Merck KGaA, Darmstadt, Germany), prior to drying at 80 °C for a day. A fine black powder was obtained at yield 15%. The yield was even higher, since a considerable amount of carbon soot escaped to open air. It could be possible to use a collecting filter similarly to flame-spray-pyrolysis for a better yield. Alternatively, but still with losses, part of the soot could be collected on a porcelain dish, likewise candle soot. The yield in this latter case was slightly higher, i.e., 18%. The whole process is illustrated in Figure 1.

At this point, we would like to emphasize that the reaction is not explosive but safe to carry out at a small scale. Indicatively, the temperature was raised from room temperature to maximum 35–40 °C after reaction. It is likely that liquid bromine acts as a coolant during reaction, removing most of the released heat by evaporation. However, most importantly, the energy value per reactants is less than 1 kJ·g^−1^ (i.e., very low as compared to fuels 40–50 kJ·g^−1^ or explosives 5–7 kJ·g^−1^). It is also important to note that air plays a significant role during the reaction course. When the reaction was run under Ar atmosphere, only a limited amount of carbon soot was released. However, a few seconds after we stopped the Ar flow and exposed the mixture in air, carbon soot started to form again. This observation merits further investigation in the near future.

The sample was macroscopically nonmagnetic (e.g., lack of magnetic iron/iron oxide phases) and nonconductive. The N_2_ specific surface area of the solid was measured at 150 m^2^·g^−1^. Based on thermal gravimetric analysis in air, combustion of the solid occurred around 350 °C and completed near 500 °C, leaving behind minor hematite residue. The iron content of the ferrocene-derived carbon was 3% w/w, the latter being in the form of hematite (α-Fe_2_O_3_). The amine-passivated derivative was obtained by reacting 50 mg of the carbon solid with 180 mg diglycolamine (HOCH_2_CH_2_OCH_2_CH_2_NH_2_, Sigma–Aldrich, St. Louis, MO, USA) at 80 °C in a small sealed glass vial for 24 h.

Powder X-ray diffraction (XRD) was performed using background-free Si wafers and Cu Ka radiation from a Bruker Advance D8 diffractometer (Bruker, Billerica, MA, USA). A RM 1000 Renishaw micro-Raman system (Renishaw, Old Town, UK) using a laser excitation line at 532 nm was used for recording the Raman spectra. For performing the thermogravimetric measurements, a Perkin Elmer Pyris Diamond TG/DTA (Perkin Elmer, Inc., Waltham, MA, USA) (TGA) was used. The sample was heated in air at a rate of 5 °C·min^−1^. X-ray photoelectron spectroscopy (XPS) measurements were carried out in an ultrahigh vacuum a SPECS GmbH spectrometer equipped with a monochromatic Mg K_α_ source (*hν* = 1253.6 eV) and a Phoibos-100 hemispherical analyzer (Berlin, Germany). The N_2_ adsorption–desorption isotherms were measured at 77 K on a Sorptomatic 1990, Thermo Finnigan porosimeter (Thermo Finnigan LLC, San Jose, CA, USA). The sample was outgassed at 120 °C for 24 h under vacuum before recording. Specific surface areas were determined with the Brunauer-Emmett-Teller (BET) method. Photoluminescence spectra were recorded using a luminescence spectrofluorometer Jasco-8300 (Tokyo, Japan), using a 1 cm path length quartz cuvette. Slit widths with a nominal band pass of 5 nm were used for both excitation and emission ray. The fluorescence emission spectra were recorded from 350 to 650 nm after excitation at different wavelengths, with a scan speed of 100 nm min^−1^. The transmission electron microscopy (TEM) study of the sample deposited on carbon coated copper grids (CF300-CU-UL, carbon square mesh, CU, 300 mesh from Electron Microscopy Science) was conducted using the instrument JEM HR-2100, JEOL Ltd., Tokyo, Japan operated at 200 kV. Atomic force microscopy (AFM) images were recorded in tapping mode with a Bruker Multimode 3D Nanoscope (Ted Pella Inc., Redding, CA, USA) using a microfabricated silicon cantilever type TAP-300G, with a tip radius of <10 nm and a force constant of approximately 20–75 N·m^−1^. Samples were suspended in ethanol by sonication (130 W, 30 min) prior to measurements.

## 3. Results and Discussion

Decades ago, the reaction between ferrocene and bromine in tetrachlorocarbon solvent (i.e., in diluted conditions) was shown to proceed via destruction of the metallocene into ferrocenium salts and pentabromocyclopentane, but not to carbon [10]. A radically different behavior was, however, observed upon direct contact of the metallocene with liquid bromine in the absence of any solvent. In this case, the reaction proceeded via rapid destruction of ferrocene into carbon, FeBr_3_, and HBr, according to Equation (1):

Fe(C_5_H_5_)_2(s)_ + 13/2 Br_2(ℓ)_ → 10 C_(s)_ + FeBr_3(s)_ + 10 HBr_(g)_, (1)

Carbon formation was evident by the naked eye (Figure 1). It is an irony, though, that ferrocene produced such a significant amount of carbon soot. For instance, ferrocene has been used instead as an additive in diesel fuel to suppress carbon soot formation (e.g., FEROX fuel tabs). On the other hand, the release of gaseous HBr was easily confirmed by the reddish coloration of a pH litmus paper placed at the top rim of the test tube. Furthermore, extraction of the crude product with water followed by filtration, gave a clear aqueous solution containing bromide and ferric ions. The bromide ions were detected through the silver nitrate test by the formation of a pale-yellow precipitate, namely silver bromide, that upon further exposure to light turned grey due to the formation of metallic silver, according to the reaction: AgBr → Ag + ½ Br_2_. In respect to the ferric ions, their presence was indicated by the formation of a characteristic blood-red colored complex with SCN^−^ ions, which disappeared on adding excess water. Apparently, the formation of carbon and trivalent iron (III) in the products is supportive of ferrocene oxidation by bromine. During oxidation of the metallocene, the cyclopentadienyl rings provided the source of carbon, whereas the Fe^2+^ ions provided the source of trivalent Fe (III).

On the basis of the molar enthalpies and entropies of the substances involved in the reaction (Table 1), we have calculated the enthalpy and entropy changes as ΔH_rxn_° = −787 kJ (exothermic) and ΔS_rxn_° = + 1 kJ/K, respectively. Hence, the free energy change at standard conditions (1 atm, 298 K) was ΔG_rxn_° = −1085 kJ < 0 (i.e., a very spontaneous reaction).

**Figure 2:**
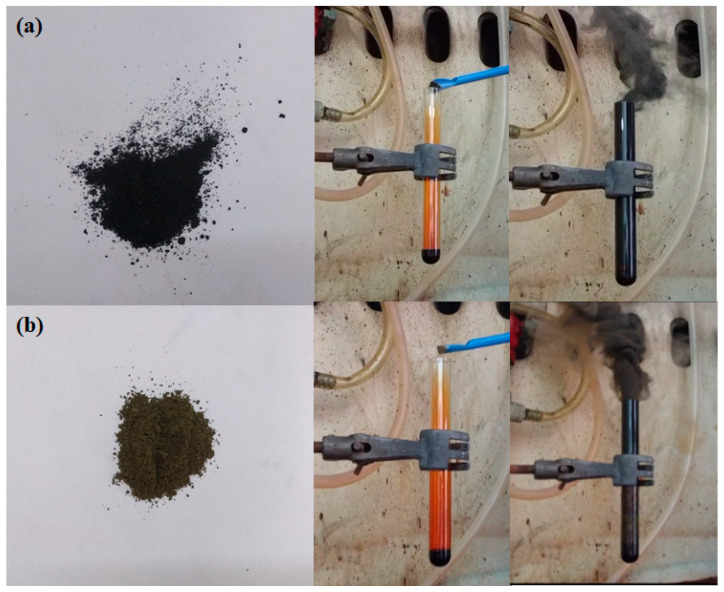
Cobaltocene (**a**) and nickelocene (**b**) reacted similarly to ferrocene upon liquid bromine to release carbon soot.

Paradoxically, the reaction was kinetically favored as well, proceeding rapidly upon contact of the reagents. This was somewhat unexpected, considering the spin change of iron as moving from diamagnetic ferrocene to paramagnetic FeBr_3_ in the reaction course. Therefore, one should rather expect a high activation energy and a slow reaction rate for this spin-forbidden reaction. Nevertheless, it must be emphasized that fast reactions involving changes in electronic spin are fairly common in transition metal chemistry, as it has been critically discussed elsewhere [11]. As the authors explicitly state in their report, the “spin forbiddenness” concept is not strictly valid in transition metals chemistry. Therefore, the fast reaction in the present case should be rather ascribed to autocatalysis by the organometallic compound, as it has been clearly suggested elsewhere [10]. Control experiments with cyclopentadienyllithium salt (C_5_H_5_^−^Li^+^), which lacks iron, gave only a minor amount of carbon soot (yield of nearly 1%), thus highlighting the catalytic role of the transition metal. In the control experiment, similar amounts of reagents as for ferrocene were used. Overall, the reaction of ferrocene with liquid bromine was both thermodynamically and kinetically favored, leading to the spontaneous and rapid formation of carbon at ambient conditions. Worth noting, the reaction was kinetically favored even at −5 °C (i.e., a couple of degrees Celsius above the normal melting point of bromine), thus making carbon synthesis viable under cold conditions as well. Lastly, it should be mentioned that cobaltocene or nickelocene fine powders worked as well as ferrocene, thus demonstrating the general character of the method (Figure 2). However, their higher price and lower chemical stability compared to ferrocene makes the latter a much more attractive starting reagent. In addition, the wealth of ferrocene derivatives available on the market today (e.g., Sigma–Aldrich) could further allow control over the composition and morphology of the derived carbons.

**Figure 3:**
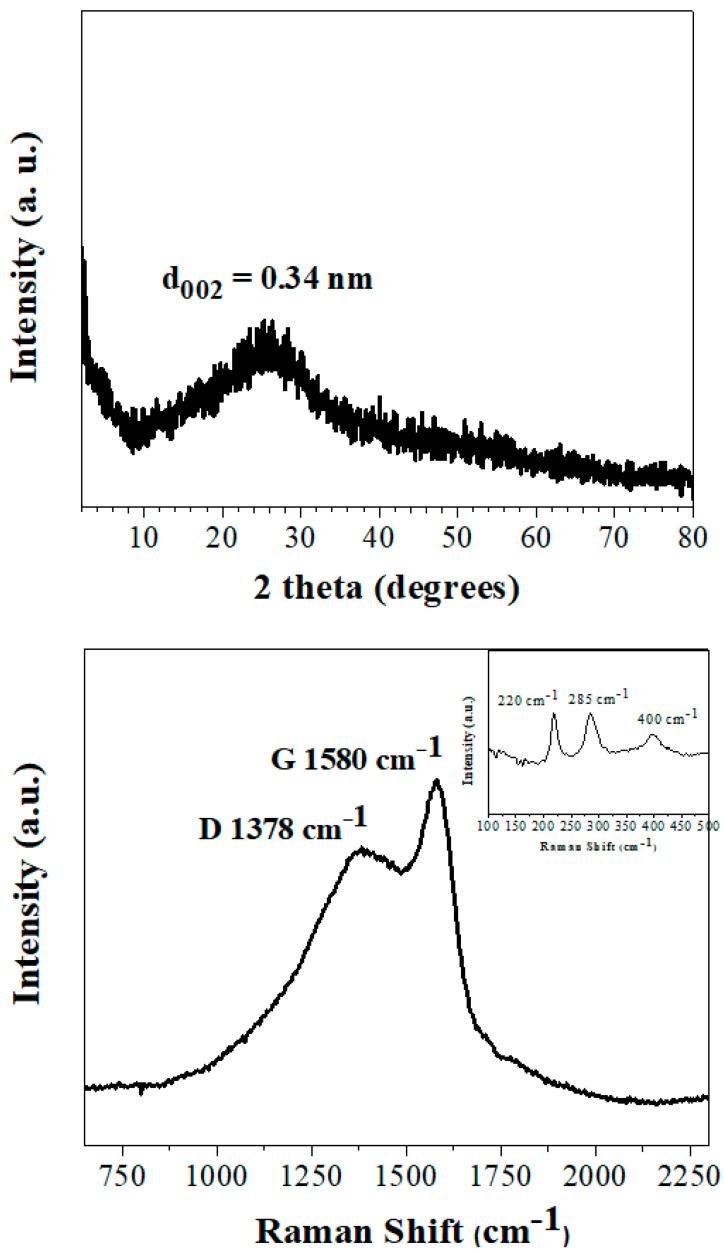
XRD pattern (**top**) and Raman spectrum (**bottom**) of the sample. The low region Raman spectrum showing the hematite peaks is given as inset.

The XRD pattern of the ferrocene-derived carbon exhibited a broad reflection at d_002_ = 3.4 Å (Figure 3, top), signaling the formation of amorphous carbon [12]. Raman spectroscopy gave the characteristic D (1378 cm^−1^) and G (1580 cm^−1^) bands with an intensity ratio of I_D_/I_G_ ~0.8 (Figure 3, bottom), thus also signaling the formation of amorphous carbon [13]. In spite of the low temperature synthesis, it is worth noting that the solid contained a significant fraction of sp^2^ carbons, as evidenced by the relatively sharper and higher intensity G band. Besides carbon, the spectrum additionally displayed characteristic low intensity peaks at 220, 285, and 400 cm^−1^ (bottom inset, Figure 3). These peaks were ascribed to hematite [14].

**Figure 4:**
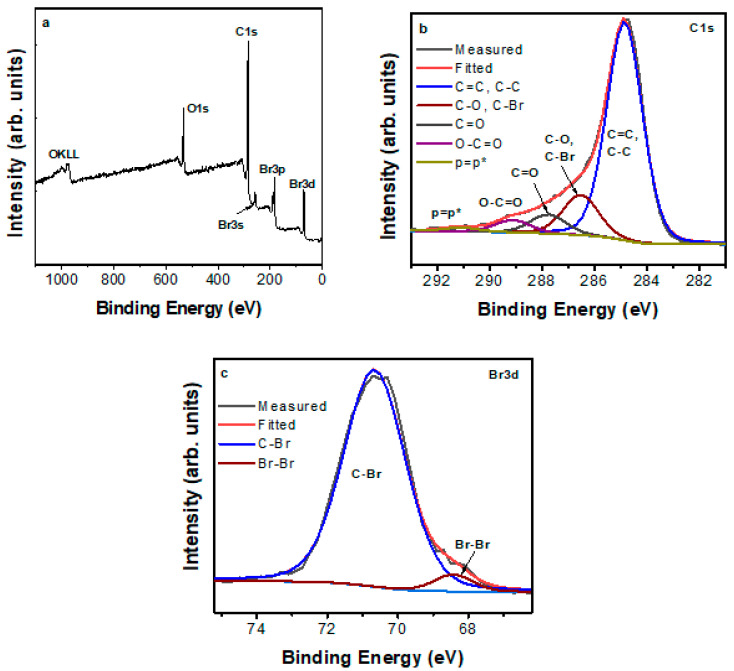
(**a**) X-ray photoelectron spectroscopy (XPS) survey spectrum and (**b**,**c**) deconvoluted C1s and Br3d high resolution spectra of the sample.

The XPS survey spectra showed the dominant presence of C (80 at. %), O (14 at. %), and Br (6 at. %) (Figure 4a). The presence of O was expected on account of synthesis in open air. The C1s spectrum (Figure 4b) was deconvoluted into five components corresponding to sp^2^/sp^3^ carbons (284.8 eV, 74.1%), C–O/C–Br (286.5 eV, 13.8%), C=O (287.8 eV, 6.5%), and O–C=O (289.1 eV, 4.3%). Note that C-O and C-Br often appear with similar binding energies [15,16]. The small component at 291.1eV (1.3%) is the satellite feature ascribed to the π–π* transition in sp^2^ conjugated carbons [17]. In Figure 4c, the high resolution Br3d spectrum was deconvoluted into two components. The main component at 70.7 eV with 95% area was attributed to the covalent C-Br bond, whereas the small one at 68.5 eV to physisorbed Br_2_ [16]. The high area of the C-Br species verified that, dominantly, Br was covalently bonded onto the carbon matrix. This bond was additionally confirmed by infrared spectroscopy by a sharp, though weak, peak at 675 cm^−1^ due to C–Br stretching.

**Figure 5:**
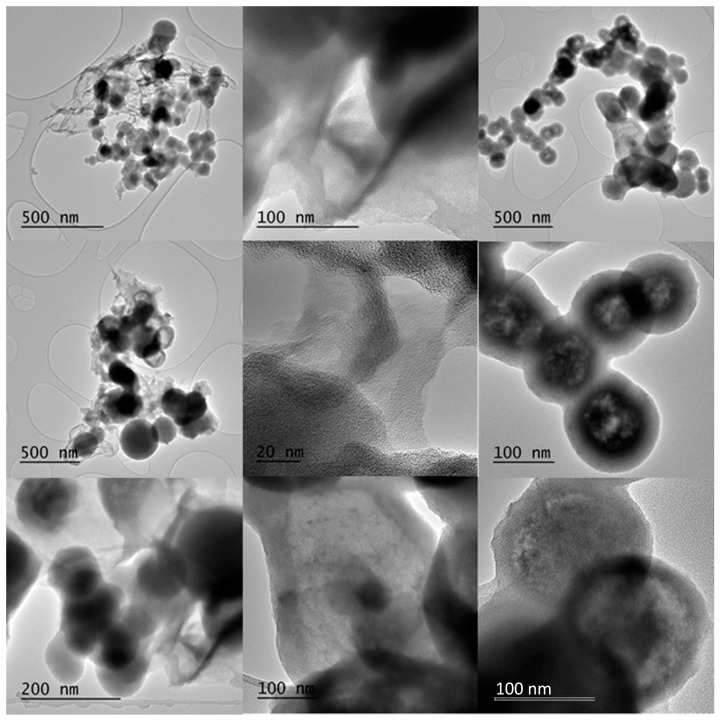
Representative TEM micrographs of carbon spheres (**left column**), nanosheets (**middle column**), and hollow spheres (**right column**).

TEM study revealed the formation of carbon spheres (diameter 50–150 nm), nanosheets (submicron to micron lateral dimensions), and hollow spheres (outer diameter 50–150 nm; wall thickness 20–30 nm) (Figure 5). Dense and hollow spheres appeared to dominate the sample, followed by nanosheets to a lesser extent. Occasionally, some tubular hollow specimens were also observed. Such features are fairly common in ferrocene-derived carbons [6,18,19].

**Figure 6:**
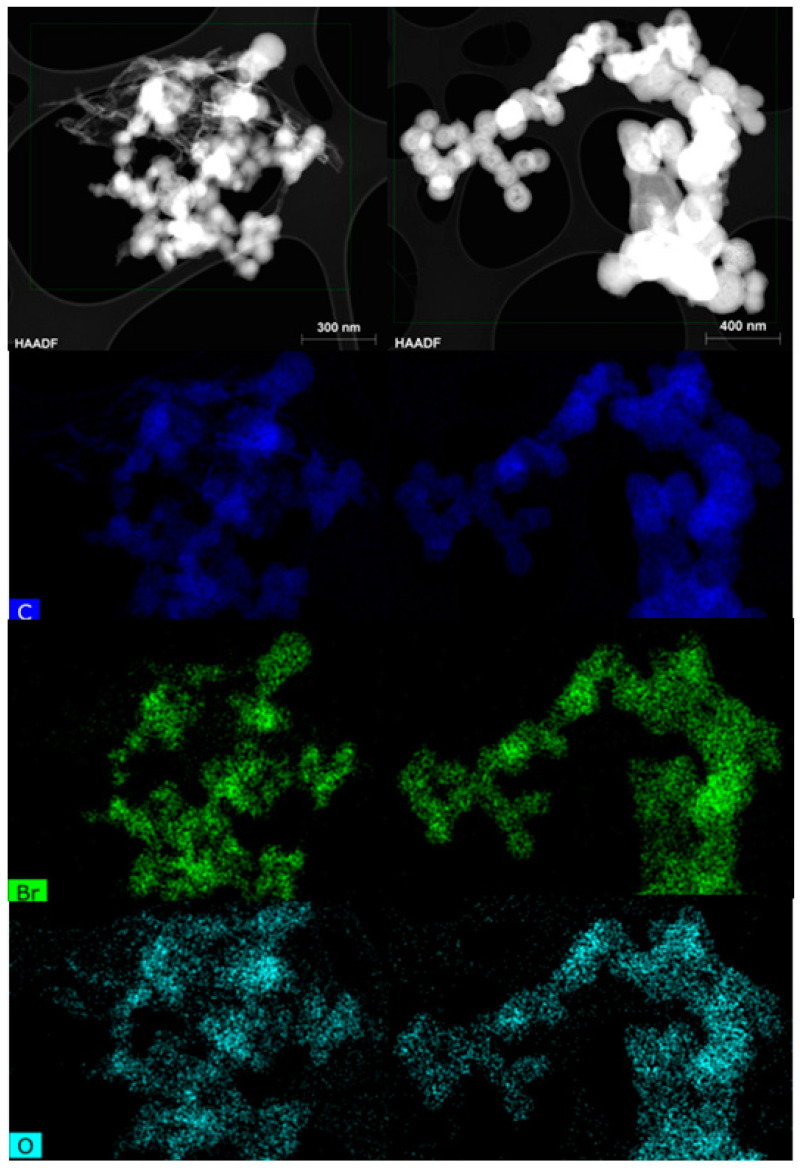
Chemical mapping of spherical particles (**left column**) and hollow spheres (**right column**). Deep blue color denotes C, green Br, and light green-blue O.

Chemical mapping showed an even distribution of the XPS-detected C, Br, and O elements in the sample (Figure 6). In other words, Br and O were uniformly distributed over the surface of the carbon matrix, thus ensuring a homogeneous composition within the sample.

**Figure 7:**
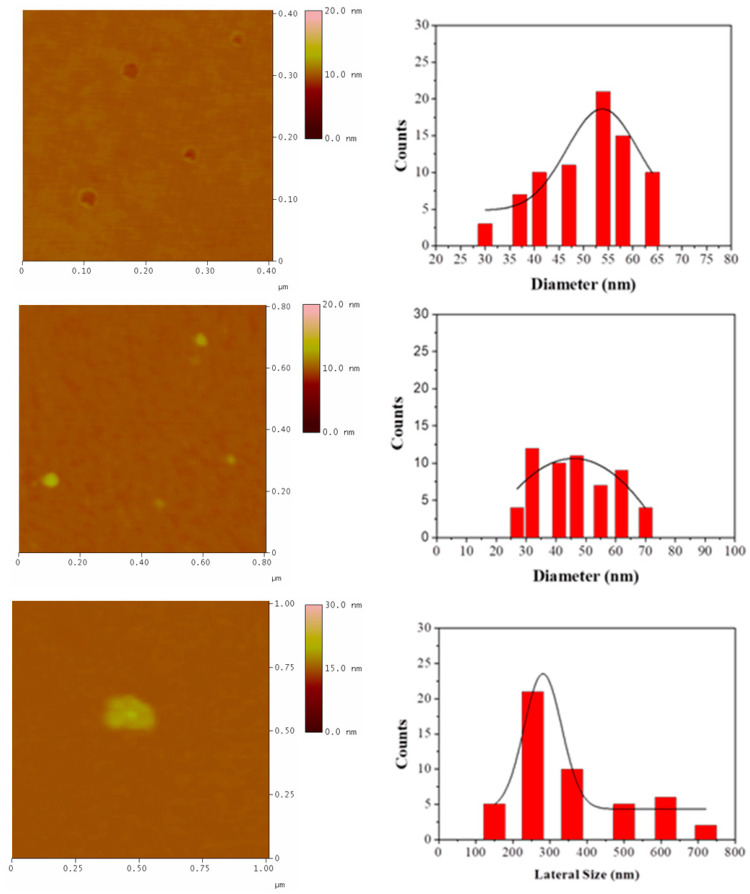
Atomic force microscopy (AFM) height images and statistical analysis of hollow spheres (**top**), dense carbon spheres (**middle**), and nanosheets (**bottom**).

To properly determine particle size distributions, we performed a statistical analysis based on AFM imaging. A total of 60 randomly selected carbon nanostructures were recorded and used for the statistical analysis histograms. The results obtained are shown in Figure 7. In the case of hollow spheres (dominating the sample), the Gaussian curve fit was centered on an average particle diameter of 54 nm. On the other hand, the diameter of the dense carbon spheres occupied a wide range between 25–71 nm. The AFM distribution histogram of the nanosheets showed that the lateral size ranged in the submicron scale.

The presence of C-Br bonds offered the possibility to modify the solid through nucleophilic substitution reactions. As an example, the bromine was replaced by the nucleophilic amine HOCH_2_CH_2_OCH_2_CH_2_NH_2_ (diglycolamine) at 80 °C, according to the reaction scheme:

C–Br + HOCH_2_CH_2_OCH_2_CH_2_NH_2_ → C–NHCH_2_CH_2_OCH_2_CH_2_OH + HBr, (2)

**Figure 8:**
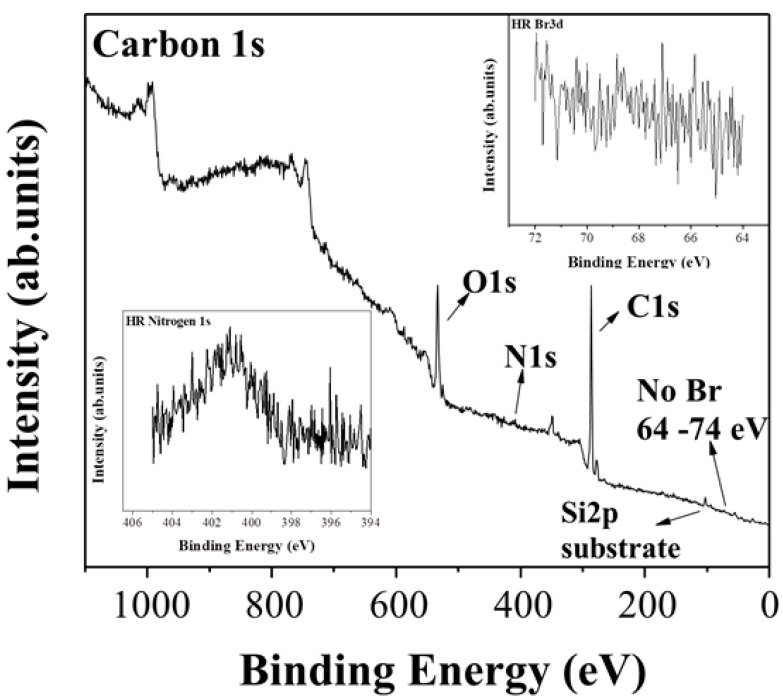
XPS survey spectrum of the amine-modified carbon. Inset top right: high resolution spectra of Br. Inset bottom left: high resolution spectra of N.

X-ray photoelectron spectroscopy (Figure 8) was applied in order to clarify the chemical functional groups of the diglycolamine-modified carbon. From the survey we can clearly observe the C1s and O1s photoelectron peaks while the peak of Br3d is absent, which is a demonstration of the nucleophilic substitution reaction. A high resolution spectra of Br (Figure 8, inset top right) is displayed showing no trace of Br in the material. The existence of nitrogen is not distinct in the survey and for this reason a high resolution spectra was applied (Figure 8, inset down left), exhibiting a clear photoelectron peak. The ratio of carbon to nitrogen was estimated at approximately 25.7 (C/N~25.7).

**Figure 9:**
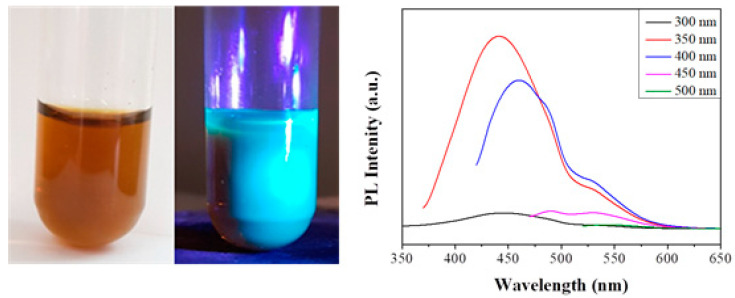
(**Left**) aqueous dispersion of the diglycolamine-modified solid under natural and UV light. (**Right**) the corresponding photoluminescence spectra of the amine-modified carbon at different excitation wavelengths (λ_ex_ as inset).

The bonded hydrophilic amine conferred aqueous dispersability to the derivative (Figure 9, left). However, most importantly, the dispersed derivative displayed bluish-green photoluminescence upon UV irradiation (Figure 9, left). This was also supported by the photoluminescence spectrum of the dispersed solid, where maximum emission was observed in the bluish-green region of the visible spectrum (Figure 9, right). The excitation-dependent emission and quantum yield (3%) of the system are typical of carbon dots [20]. Note that such properties were not displayed by the pristine solid or diglycolamine. In general, the quantum yield of carbon dots is lower than that of conventional quantum dots, ranging between 3–10% vs. 50%. Much higher values are rather considered suspicious in terms of purity of the carbon dots [21].

As described elsewhere, surface passivation is a fundamental photoluminescence-enhancing tool for improving emission from poor carbon emitters and usually involves amines as passivation agents [22,23,24,25,26]. Apart from this, it is also important to mention the role of diglycolamine as modifier. For instance, the amine is commonly used as a linker in the synthesis of bioconjugate materials for applications in drug delivery through hydrogen bonding interactions [27]. All these features combined make the fluorescent derivative promising in bioapplications (e.g., bioimaging, cell labeling, and drug delivery). From the abovementioned, it becomes apparent that proper surface engineering of the carbon particles may impart new properties and functionalities for various applications.

## 4. Conclusions

We have presented a rapid and spontaneous carbon synthesis from ferrocene without pyrolysis. The method was merely based on reacting ferrocene with liquid bromine at ambient conditions by simply bringing them into contact. The derived carbon was mainly composed of dense spherical particles, nanosheets, and hollow spheres. The presence of reactive C–Br surface groups permitted further functionalization of the particles, as for instance with diglycolamine. The obtained derivative was dispersible in aqueous solvents and fluoresced in the visible region due to emission from a passivated surface. This result showed how surface modification through pending functionalities could further tailor the physical properties of the solid. Altogether, spontaneity at ambient conditions, rapidity, easy surface modification, and wider applicability to other metallocenes make the present method attractive in carbon materials synthesis as complementary to hypergolic reactions.

## Figures and Tables

**Table 1:** Molar enthalpy (H°) and entropy (S°) values of the involved chemicals at standard conditions (1 atm, 298 K).

Compound	Fe(C_5_H_5_)_2(s)_	Br_2(ℓ)_	C_(s)_	FeBr_3(s)_	HBr_(g)_
			(Graphite)		
H° (kJ·mol^−1^)	158	0	0	−269	−36
S° (kJ·K^−1^·mol^−1^)	0.216	0.152	0.00574	0.184	0.2

## References

[1] Georgakilas V., Perman J.A., Tucek J., Zboril R. (2015). Broad family of carbon nanoallotropes: Classification, chemistry, and applications of fullerenes, carbon dots, nanotubes, graphene, nanodiamonds, and combined superstructures. Chem. Rev..

[2] Baikousi M., Chalmpes N., Spyrou K., Bourlinos A.B., Avgeropoulos A., Gournis D., Karakassides M.A. (2019). Direct production of carbon nanosheets by self-ignition of pyrophoric lithium dialkylamides in air. Mater. Lett..

[3] Chalmpes N., Spyrou K., Bourlinos A.B., Moschovas D., Avgeropoulos A., Karakassides M.A., Gournis D. (2020). Synthesis of highly crystalline graphite from spontaneous ignition of in situ derived acetylene and chlorine at ambient conditions. Molecules.

[4] Chalmpes N., Asimakopoulos G., Spyrou K., Vasilopoulos K.C., Bourlinos A.B., Moschovas D., Avgeropoulos A., Karakassides M.A., Gournis D. (2020). Functional carbon materials derived through hypergolic reactions at ambient conditions. Nanomaterials.

[5] Chalmpes N., Spyrou K., Vasilopoulos K.C., Bourlinos A.B., Moschovas D., Avgeropoulos A., Gioti C., Karakassides M.A., Gournis D. (2020). Hypergolics in carbon nanomaterials synthesis: New paradigms and perspectives. Molecules.

[6] Hou H., Schaper A.K., Weller F., Greiner A. (2002). Carbon nanotubes and spheres produced by modified ferrocene pyrolysis. Chem. Mater..

[7] Hu Z.D., Hu Y.F., Chen Q., Duan X.F., Peng L.M. (2006). Synthesis and characterizations of amorphous carbon nanotubes by pyrolysis of ferrocene confined within AAM templates. J. Phys. Chem. B.

[8] Riquelme J., Garzón C., Bergmann C., Geshev J., Quijada R. (2016). Development of multifunctional polymer nanocomposites with carbon-based hybrid nanostructures synthesized from ferrocene. Eur. Polym. J..

[9] Su W., He M., Xing J., Zhong Y., Li Z. (2013). Facile synthesis of porous bifunctional Fe_3_O_4_@Y_2_O_3_:Ln nanocomposites using carbonized ferrocene as templates. RSC Adv..

[10] Nesmeyanov A.N., Anisimov K.N., Kolobova N.E., Zlotina I.B. (1968). Action of bromine and chlorine on cyclopentadienylmanganesetricarbonyl. Bull. Acad. Sci. USSR Div. Chem. Sci..

[11] Poli R., Harvey J.N. (2003). Spin forbidden chemical reactions of transition metal compounds. New ideas and new computational challenges. Chem. Soc. Rev..

[12] Majewska J., Michalkiewicz B. (2013). Low temperature one-step synthesis of cobalt nanowires encapsulated in carbon. Appl. Phys. A.

[13] Roh J.S. (2008). Structural study of the activated carbon fiber using laser Raman spectroscopy. Carbon Lett..

[14] Mansour H., Letifi H., Bargougui R., De Almeida-Didry S., Negulescu B., Autret-Lambert C., Gadri A., Ammar S. (2017). Structural, optical, magnetic and electrical properties of hematite (α-Fe_2_O_3_) nanoparticles synthesized by two methods: Polyol and precipitation. Appl. Phys. A.

[15] Jankovský O., Šimek P., Klimová K., Sedmidubský D., Matějková S., Pumera M., Sofer Z. (2014). Towards graphene bromide: Bromination of graphite oxide. Nanoscale.

[16] Zheng J., Liu H.T., Wu B., Di C.A., Guo Y.L., Wu T., Yu G., Liu Y.Q., Zhu D.B. (2012). Production of graphite chloride and bromide using microwave sparks. Sci. Rep..

[17] Xie W., Ng K.M., Weng L.T., Chan C.M. (2016). Characterization of hydrogenated graphite powder by X-ray photoelectron spectroscopy and time-of-flight secondary ion mass spectrometry. RSC Adv..

[18] Zou G., Yu D., Lu J., Wang D., Jiang C., Qian Y. (2004). A self-generated template route to hollow carbon nanospheres in a short time. Solid State Commun..

[19] Boi F.S., Guo J., Medranda D., Borowiec J., Liu D., Wang S., Zhang X., He Y., Xiang G. (2018). Observation of curling effects in tubular and planar graphene-like structures by pyrolysis of ferrocene/dichlorobenzene mixtures. Mater. Today Chem..

[20] Bourlinos A.B., Zbořil R., Petr J., Bakandritsos A., Krysmann M., Giannelis E.P. (2012). Luminescent surface quaternized carbon dots. Chem. Mater..

[21] Essner J.B., Kist J.A., Polo-Parada L., Baker G.A. (2018). Artifacts and errors associated with the ubiquitous presence of fluorescent impurities in carbon nanodots. Chem. Mater..

[22] Li L., Dong T. (2018). Photoluminescence tuning in carbon dots: Surface passivation or/and functionalization, heteroatom doping. J. Mater. Chem. C.

[23] Potsi G., Bourlinos A.B., Mouselimis V., Poláková K., Chalmpes N., Gournis D., Kalytchuk S., Tomanec O., Błoński P., Medveď M. (2019). Intrinsic photoluminescence of amine-functionalized graphene derivatives for bioimaging applications. Appl. Mater. Today.

[24] Das R., Bandyopadhyay R., Pramanik P. (2018). Carbon quantum dots from natural resource: A review. Mater. Today Chem..

[25] Chu K.W., Lee S.L., Chang C.J., Liu L. (2019). Recent progress of carbon dot precursors and photocatalysis applications. Polymers.

[26] Wang X., Feng Y., Dong P., Huang J. (2019). A mini review on carbon quantum dots: Preparation, properties, and electrocatalytic application. Front. Chem..

[27] 2-(2-Aminoethoxy)ethanol. https://www.sigmaaldrich.com/catalog/product/aldrich/a54059?lang=en&region=GR.

